# Minimally invasive endoscopic treatment for lumbar infectious spondylitis: a retrospective study in a tertiary referral center

**DOI:** 10.1186/1471-2474-15-105

**Published:** 2014-03-27

**Authors:** Shih-Chieh Yang, Tsai-Sheng Fu, Hung-Shu Chen, Yu-Hsien Kao, Shang-Won Yu, Yuan-Kun Tu

**Affiliations:** 1 Department of Orthopaedic Surgery and Anesthesiology, E-Da Hospital, I-Shou University, Kaohsiung, Taiwan; 2 Department of Orthopaedic Surgery, Chang Gung Memorial Hospital, Taoyuan, Taiwan

**Keywords:** Betadine, Endoscopic debridement, Infectious spondylitis, Minimally invasive surgery

## Abstract

**Background:**

Spinal infections remain a challenge for clinicians because of their variable presentation and complicated course. Common management approaches include conservative administration of antibiotics or aggressive surgical debridement. The purpose of this study was to evaluate the efficacy of percutaneous endoscopic debridement with dilute betadine solution irrigation (PEDI) for treating patients with lumbar infectious spondylitis.

**Methods:**

From January 2005 to July 2010, a total of 32 patients undergoing PEDI were retrospectively enrolled in this study. The surgical indications of the enrolled patients included single-level infectious spondylodiscitis, postoperative infectious spondylodiscitis, advanced infection with epidural abscess, psoas muscle abscess, pre-vertebral or para-vertebral abscess, multilevel infectious spondylitis, and recurrent infection after anterior debridement and fusion. Clinical outcomes were assessed by careful physical examination, Macnab criteria, regular serologic testing, and imaging studies to determine whether continued antibiotics treatment or surgical intervention was required.

**Results:**

Causative bacteria were identified in 28 (87.5%) of 32 biopsy specimens. Appropriate parenteral antibiotics for the predominant pathogen isolated from infected tissue biopsy cultures were prescribed to patients. Twenty-seven (84.4%) patients reported satisfactory relief of their back pain after PEDI. Twenty-six (81.3%) patients recovered uneventfully after PEDI and sequential antibiotic therapy. No surgery-related major complications were found, except 3 patients with transient paresthesia in the affected lumbar segment.

**Conclusions:**

PEDI was successful in obtaining a bacteriologic diagnosis, relieving the patient’s symptoms, and assisting in the eradication of lumbar infectious spondylitis. This procedure could be an effective alternative for patients who have a poor response to conservative treatment before a major open surgery.

## Background

Spinal infections continue to be a considerable diagnostic and therapeutic challenge to clinicians because of their subtle presentation in the early stages. Medical imaging technology, laboratory tests, and a high degree of suspicion can help spinal surgeons make a correct diagnosis in a timely fashion to avoid severely debilitating complications [1-4]. Identifying the offending pathogen is critical to provide appropriate antibiotic therapy for medical treatment. Computer tomography-guided needle biopsy was previously recommended for isolating causative pathogens [5-7]. However, the aspirate was often inadequate and sometimes no organism was cultured. A delay in appropriate treatment for spinal infections can lead to structure instability, spinal deformity, sepsis, neurologic deficit, and even death.

Percutaneous endoscopic discectomy (PED) was first employed for treating uncomplicated herniated discs in the early 1980s [8]. Recently, numerous minimally invasive percutaneous endoscopic techniques for lumbar disc herniation and even spinal stenosis have been developed, with clinical outcomes comparable to those of conventional open surgery [9,10]. Some studies also reported that PED could be applied in the management of spinal infections [11-13]. Povidone-iodine (betadine) is a widely used antiseptic and disinfectant agent. It can eradicate most pathogens, including oxacillin resistant staphylococcus aureus, and no bacterial resistance has been reported [14-18]. Additionally, a lower concentration of 0.5% betadine has few cytotoxic effects [19,20]. The purpose of this study was to evaluate the diagnostic and therapeutic efficacy of percutaneous endoscopic debridement with dilute betadine solution irrigation (PEDI) in the treatment of patients with infectious spondylitis.

## Methods

### Patients

This study was approved by the Institutional Review Board of E-DA Hospital, a 1200-bed tertiary referral center (IRB number: EMPR-101-027). Thirty-two patients with infectious spondylitis underwent PEDI procedures between January 2005 and July 2010 at our institute. There were 9 women and 23 men with an average age of 57.4 years (range, 38-88 years). The patients’ medical records, including outpatient and emergency room notes, admission notes, inpatient progress and nursing notes, discharge summaries, procedure notes, surgical reports, radiology reports, pathology reports, and microbiology laboratory results were reviewed. The microbiology reports comprised microscopy and culture findings and any specific pathogens identified by either method. All of the patients presented with intractable back pain requiring narcotic pain control and bed rest. Infectious spondylitis was diagnosed on the basis of clinical examinations, including elevated erythrocyte sedimentation rate (ESR) and C-reactive protein (CRP) values, and radiographic and magnetic resonance imaging (MRI) findings.

The surgical indications of the 32 patients undergoing PEDI included single-level infectious spondylodiscitis, postoperative infectious spondylodiscitis, advanced infection with epidural abscess, psoas muscle abscess, pre-vertebral or para-vertebral abscess, multilevel infectious spondylitis, and recurrent infection after anterior debridement and fusion. The infectious spondylitis of all patients who underwent PEDI in this study did not respond to conservative administration of empirical antibiotics and was not so severe to absolutely need aggressive surgical debridement. Patients who sustained severe infection resulting in severe structural deformity or significant neurological deficit were excluded in this study and they were advised to receive open surgery for their severe infectious spondylitis.

### Intervention

**Figure 1:**
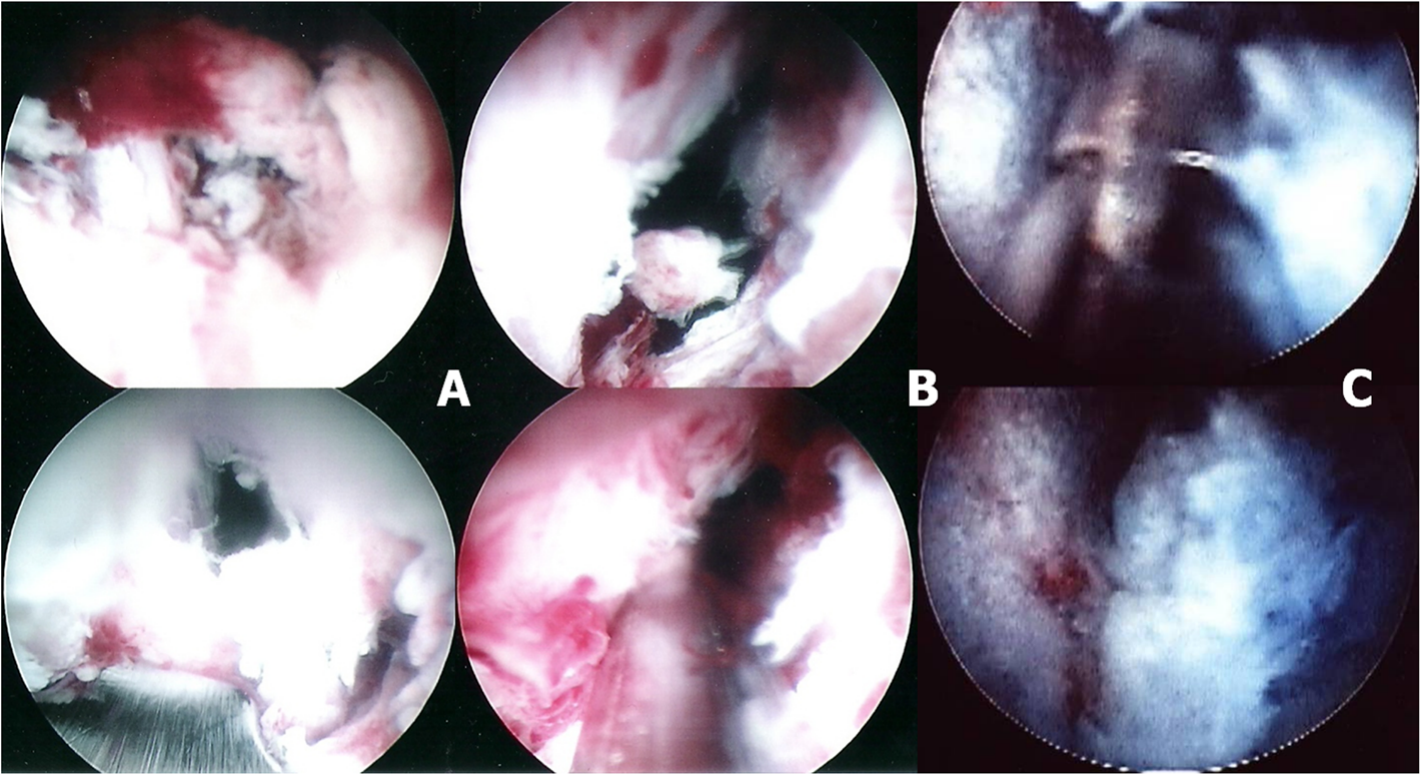
**Intraoperative endoscopic views from the inside of L5-S1 infectious spondylitis.** At the beginning of the PEDI procedure, the endoscopic view was not clear due to pus accumulation and granulation tissue at the infected disc level **(A)**. Discectomy forcep, flexible rongeur, and shaver were then inserted through the cannulated sleeve to withdraw as much infected tissue as possible **(B)**. By aggressive percutaneous debridement, the endoscopic view became much clear and the vertebral endplate above the infected disc level could be identified **(C)**.

The patient was placed prone on a radiolucent frame suitable for fluoroscopy. All procedures were performed under local anesthesia with conscious sedation similar to that used for standard lumbar discography. Under fluoroscopic guidance, the target site was located and the entry site was marked on the skin at a point 8–12 cm from the midline. After sterile preparation, draping, and local anesthesia, a spinal needle was inserted directly into the center of the target disc. Conscious sedation was performed through administering IV bolus dose of fentanyl 50 mcg and propofol 0.5 mg · kg^-1^. A guide wire was introduced through the spinal needle into the central disc space, and the spinal needle was then withdrawn. After creating a small stab-wound incision (about 1 cm), a dilator and a cannulated sleeve were guided over the wire and progressed sequentially into the disc center. Additional IV bolus dose of propofol 0.5 mg · kg^-1^ were administered if the patients complained pain during the procedures. Fluoroscopy was repeated in 2 orthogonal planes to verify the correct position of the endoscope tip. The tissue dilator was then removed, and the cutting tool was inserted. The cutting tool, a cylindrical sleeve with a serrated edge at its distal end, was used to harvest a core of the biopsy specimen. Discectomy forceps were then inserted through the cannulated sleeve to extract additional tissue from the infected disc. Percutaneous debridement was conducted piecemeal by manipulating the biopsy forceps, flexible rongeurs, and shaver into different positions to withdraw as much tissue as possible under fluoroscopic monitoring and endoscopic view (Figure 1). The same procedures were repeated on the other side. These 2 working sheaths were left on both sides for sufficient extirpation and extensive debridement of the infected intervertebral disc and even some endplate from different endoscopic direction.

**Figure 2:**
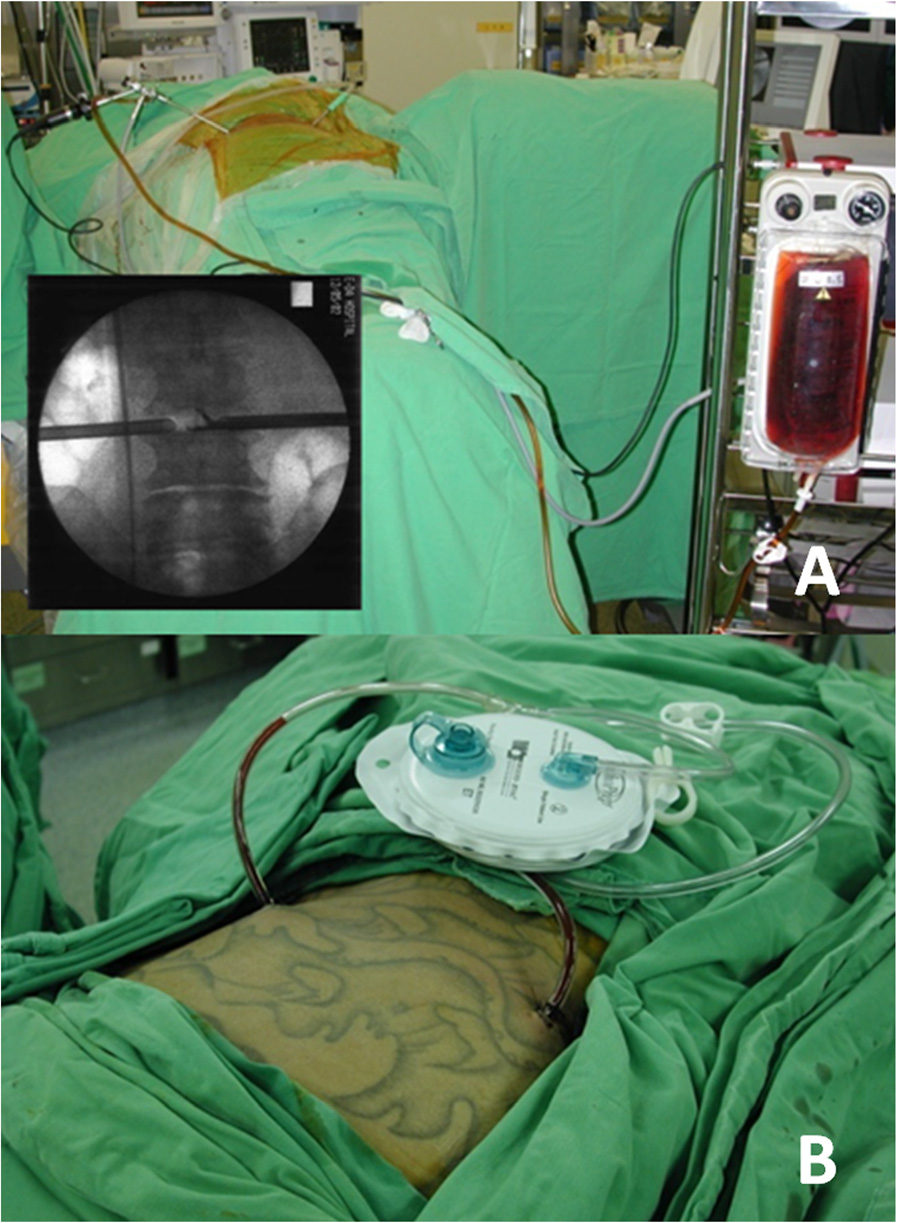
**PEDI procedure.** Two portal working sheaths were used for percutaneous endoscopic debridement followed by dilute betadine solution irrigation (PEDI) for treating patients with lumbar infectious spondylitis **(A)**. A negative-pressure Hemovac with 2 drainage tubes was inserted through the sheaths for further continuous drainage of the offending pathogens **(B)**. Informed consent was obtained from the patient for his image to be published in this study.

After the biopsy and debridement procedures, at least 10,000 mL of dilute betadine solution was used for irrigation. Approximately 35 mL of povidone-iodine was diluted with 1,000 mL normal saline to achieve a 0.35% betadine solution for use during the operation. One portal was connected to a lavage fluid pump for the inflow and the other portal was connected to a suction bottle for the outflow, with continuous infusion (Figure 2). The suction function is usually kept open from the beginning to the end of the procedure for further drainage of the lavage fluid and abscess. Finally, 2 drainage tubes were inserted into the debrided disc space and connected to a negative-pressure Hemovac. The biopsy specimen contained disc material and parts of vertebral endplates of adjacent vertebrae. Each biopsy specimen was examined for microorganisms and evaluated histopathologically.

### Outcome measures

Clinical outcomes were assessed by careful physical examination, Macnab criteria, regular serological tests, and imaging studies during admission, at 1 month after discharge and every 3 months to determine whether continued conservative treatment was sufficient or open surgical intervention was required [21]. All patients were followed up for at least 24 months after undergoing PEDI treatment.

## Results

### Patients

All 32 patients met the inclusion criteria, and their medical records were analyzed in this study. Table 1 presents a summary of these patients’ data, including diagnosis and involved level, offending pathogens, and clinical outcome after PEDI. Fifteen patients with single-level infection, 4 with multilevel infection (3 with 2-level infection and 1 with 3-level infection), 5 with postoperative infection, 5 with paraspinal abscess, and 3 with epidural abscess were enrolled in this study. The most prominent clinical sign of infectious spondylitis was back pain, which was detected in all 32 patients before PEDI. One week after PEDI, 2 patients had excellent outcomes, 25 had good outcomes, 4 had fair outcomes, and 1 had a poor outcome according to Macnab criteria. A total of 27 patients (84.4%) reported satisfactory relief of their back pain after PEDI. Of these 27 patients, 2 experienced recurrent intractable pain with progressive kyphotic deformity identified radiologically, and underwent surgical treatment at 2 and 5 months after PEDI, respectively. Four of the remaining 5 patients who had persistent infection and severe back pain after PEDI underwent anterior debridement accompanied by autograft interbody fusion within 2 weeks after PEDI. Among these 4 patients, 1 had a single-level infection and the other 3 had advanced infection with multilevel involvement.

**Table 1:** Patients’ demographic data and clinical outcomes

**Case no.**	**Diagnosis**	Macnab criteria*	**Culture**	**Open surgery**	**Complication**
1	L2-3 single-level infection	Good	OSSA	None	None
2	L3-4 postoperative infection	Good	ORSA	None	None
3	L2-3 single-level infection	Good	No growth	None	None
4	L2-3 single-level infection	Good	ORSA	2 months later	None
5	L3-4 infection with paraspinal abscess	Good	Pseudomonas aeruginosa	None	None
6	L5-S1 single-level infection	Good	Prevotella	None	None
7	L5-S1 infection with epidural abscess	Good	Streptococcus viridans	None	None
8	L3-4, L4-5, and L5-S1 multilevel infection	Poor	Mycobacterium tuberculosis	1 week later	Paresthesia
9	L4-5 and L5-S1 multilevel infection	Good	ORSA	None	None
10	L2-3 single-level infection	Good	ORSA, Haemophilus influenzae	None	None
11	L4-5 single-level infection	Good	OSSA	None	None
12	L1-2 single-level infection	Good	Enterococcus faecalis	None	None
13	L3-4 and L4-5 multilevel infection	Fair	Pseudomonas aeruginosa	2 weeks later	Paresthesia
14	L2-3 infection with paraspinal abscess	Good	OSSA	None	None
15	L4-5 postoperative infection	Good	Streptococcus viridans	None	None
16	L2-3 single-level infection	Good	Haemophilus influenzae	None	None
17	L4-5 single-level infection	Excellent	OSSA	None	None
18	L5-S1 infection with epidural abscess	Good	ORSA	None	None
19	L3-4 postoperative infection	Good	OSSA	None	None
20	L2-3 single-level infection	Fair	No growth	1 week later	None
21	L5-S1 postoperative infection	Good	OSSA	None	None
22	L5-S1 single-level infection	Good	No growth	None	None
23	L4-5 single-level infection	Fair	No growth	None	None
24	L4-5 infection with epidural abscess	Good	OSSA, Escherichia coli	None	None
25	L3-4 and L4-5 multilevel infection	Fair	Haemophilus influenza	2 weeks later	Paresthesia
26	L5-S1 infection with presacral abscess	Good	ORSA	None	None
27	L4-5 single-level infection	Good	klebsiella pneumoniae	None	None
28	L4-5 single-level infection	Good	Candida albicans	None	None
29	L3-4 infection with paraspinal abscess	Good	OSSA	5 months later	None
30	L2-3 infection with paraspinal abscess	Excellent	ORSA	None	None
31	L4-5 postoperative infection	Good	ORSA	None	None
32	L4-5 single-level infection	Good	OSSA	None	None

M = male, F = female, L = lumbar spine, S = sacral spine, OSSA = oxacillin sensitive staphylococcus aureus, ORSA = oxacillin resistant staphylococcus aureus.

*Evaluation at one week after PEDI.

### Isolated pathogens

Causative bacteria were isolated in 28 (87.5%) of 32 infected tissue biopsy cultures. Of these 28 patients, 2 patients had 2 different pathogens cultured. Seventeen patients were infected with staphylococcus aureus, 8 with the oxacillin resistant strain and 9 with the oxacillin sensitive strain. Three patients had haemophilus influenzae infection, 2 had streptococci viridans, 2 had pseudomonas aeruginosa, and the other 6 had prevotella species, mycobacterium tuberculosis, enterococcus faecalis, escherichia coli, klebsiella pneumoniae, and candida albicans infection, respectively. Systemic antibiotics and anti-tuberculous or anti-fungal chemotherapy were administered according to sensitivity studies for identified pathogens. There were no pathogens isolated from the other 4 patients (12.5%). Two of them had good outcomes after PEDI, but the other 2 had persistent back pain. Short-term broad-spectrum antibiotics were administrated after PEDI in these patients. Three of the 4 patients recovered uneventfully after treatment with broad-spectrum antibiotics. The other one underwent surgical intervention for intractable back pain and was proved to have staphylococcus aureus infection by open surgical biopsy.

### Clinical outcomes

**Figure 3:**
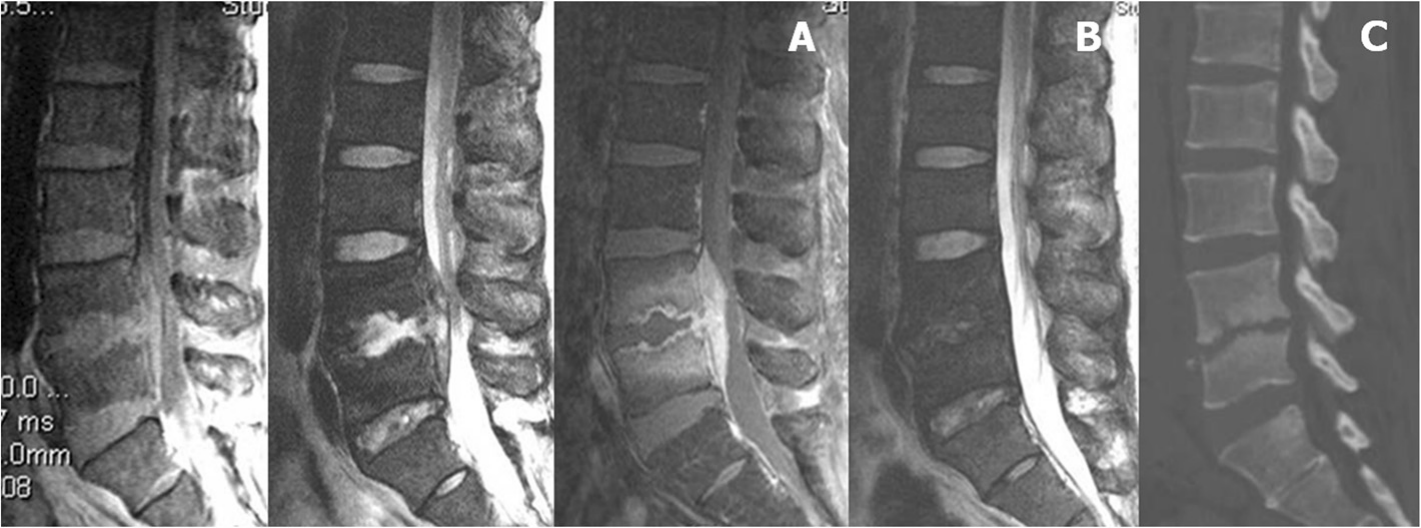
**Epidural abscess treated by PEDI.** L4-5 infectious spondylitis was diagnosed in a male patient. Sagittal T1- and T2-weighted and contrast magnetic resonance imaging (MRI) showed L4-5 epidural abscess with compression of neural elements **(A)**. After PEDI treatment, sagittal T2-weighted MRI at 6 months follow-up demonstrated the disappearance of the abscess **(B)**. Lateral computed tomograph revealed L4-5 disc space narrowing leading to spontaneous fusion **(C)**.

**Figure 4:**
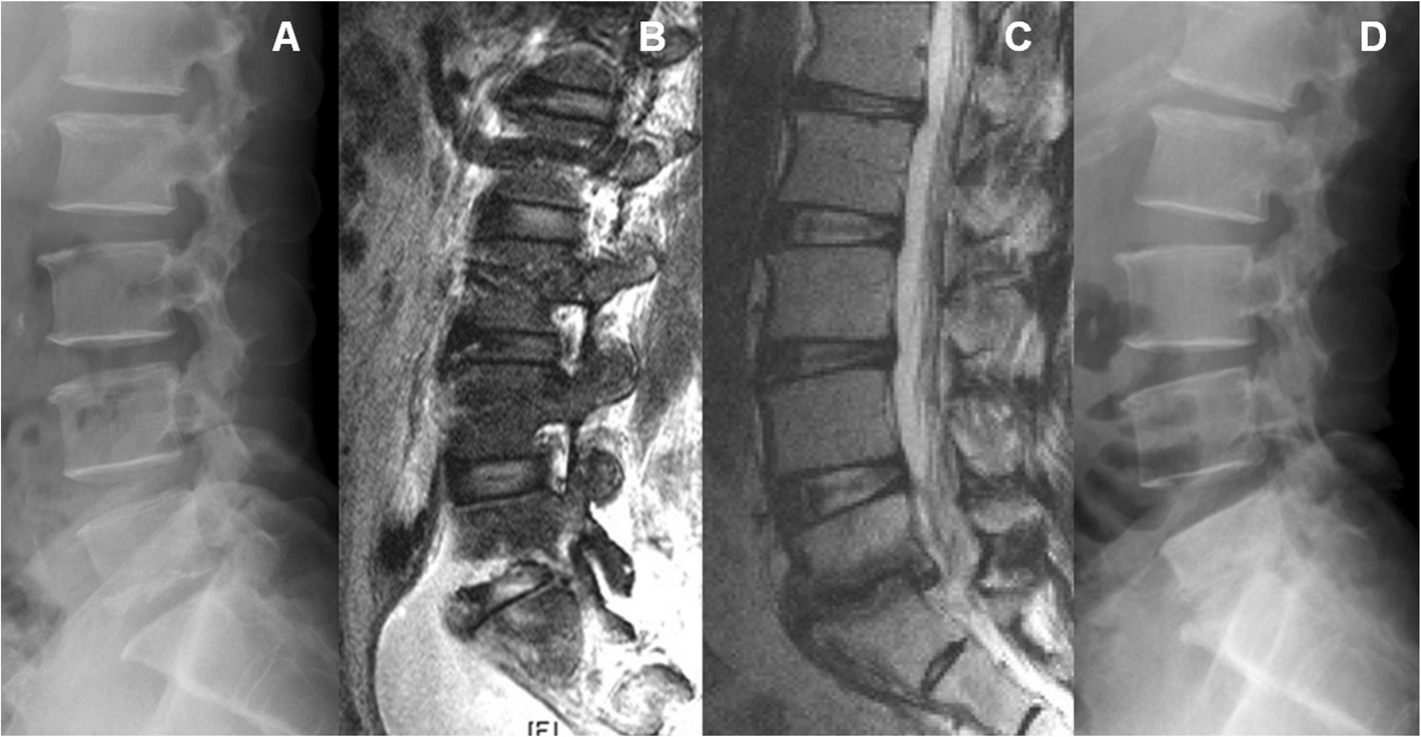
**Presacral abscess treated by PEDI.** L5-S1 infectious spondylitis was diagnosed in a male patient. The lateral radiograph showed L5 inferior endplate erosion **(A)**. Sagittal T2-weighted MRI demonstrated presacral abscess **(B)**. Postoperative sagittal T2-weighted MRI revealed the abscess was eradicated by PEDI **(C)**. Postoperative lateral radiograph showed L5-S1 disc space collapse leading to spontaneous fusion **(D)**.

**Figure 5:**
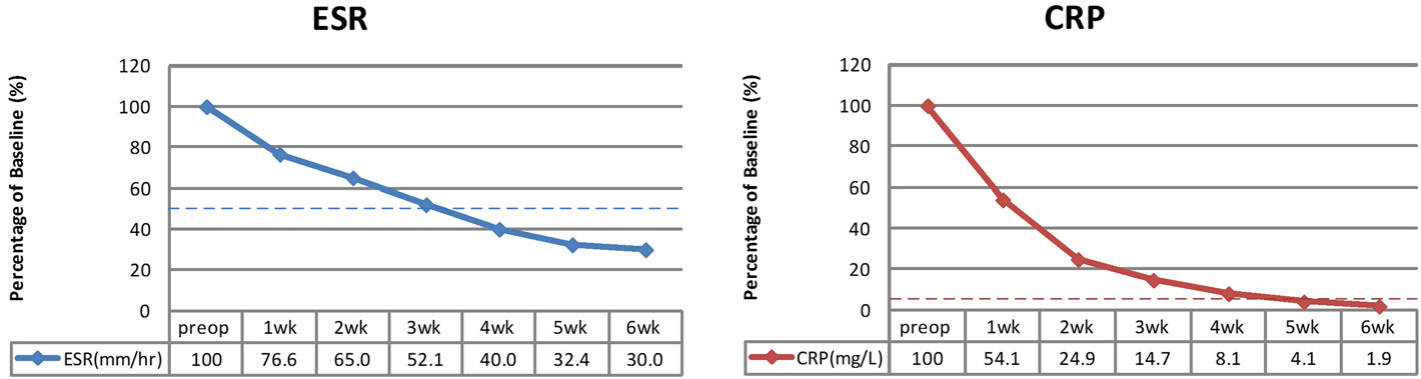
**ESR and CRP changed by PEDI.** Percentage changes in serological values, before and after PEDI, in successfully treated patients.

Overall, 6 of 32 patients underwent anterior debridement and autograft interbody fusion with iliac crest because of persistent severe back pain, failure to identify the pathogens, or progressive infection. In addition to 2 patients with single-level infection, 1 with paraspinal abscess and the other 3 with multilevel infections were included. Three patients with multilevel infections who had uncontrolled infection and mechanical instability underwent anterior debridement and autograft interbody fusion, followed by supplemental posterior fixation. Extensive osteolytic destruction of the vertebral body with spinal instability or kyphotic deformity was observed in these 6 patients. Twenty-six (81.3%) patients responded to PEDI and were successfully treated with at least a 6-week course of parenteral antibiotics therapy or full-course antimicrobial chemotherapy (Figures 3 and 4). The changes in serological values before and after PEDI in the 26 successfully treated patients are shown in Table 2. Elevated CRP values returned to reference ranges with a mean period of 4.2 weeks in these patients, whereas elevated ESR irregularly decreased to half of the original pretreatment values within a mean period of 3.4 weeks (Figure 5). White blood cell count was elevated only in 10 (38.5%) of these patients and seemed to be a relatively poor indicator for spinal infection. No recurrent infection was found among these patients during at least 24 months of follow-up (average, 38.5 months; range, 24-60 months). No surgery-related major complications were noted, except in 3 patients who complained of transient paresthesia in the affected lumbar segment.

**Table 2:** Highest preoperative Erythrocyte Sedimentation Rate (ESR), C-reactive Protein (CRP), and White Blood Cell (WBC) count, and time to return to normal value in 26 patients successfully treated by percutaneous endoscopic debridement with dilute betadine solution irrigation

**Case no.**	**Highest preop ESR (mm/h)**	**time to half of original Preop value (weeks)**	**Highest preop CRP (mg/L)**	**Time to normal value (weeks)**	**Highest preop WBC (/μL)**	**Time of abnormal WBC to normal value (weeks)**
1	83	3	112	4	8960	Initial WNL
2	18	4	25	3	7670	Initial WNL
3	120	3	102	5	9990	Initial WNL
4	55	2	31	2	11030	3
5	50	5	78	4	7520	Initial WNL
6	55	3	46	4	8630	Initial WNL
7	34	4	22	2	8660	Initial WNL
8	34	1	138	4	12370	3
9	86	5	104	6	9640	Initial WNL
10	33	4	38	4	6920	Initial WNL
11	52	4	103	3	10140	Initial WNL
12	55	3	46	3	16140	4
13	131	6	35	4	12370	3
14	49	4	38	2	5780	Initial WNL
15	60	4	93	6	4630	Initial WNL
16	111	4	104	6	16490	5
17	104	5	182	6	21620	5
18	34	3	18	2	9190	Initial WNL
19	37	5	177	6	7820	Initial WNL
20	58	3	64	2	11130	2
21	39	2	58	6	11770	3
22	67	1	93	3	3590	Initial WNL
23	123	1	377	6	14990	4
24	62	2	92	6	6590	Initial WNL
25	37	3	52	6	8750	Initial WNL
26	62	4	89	5	12690	3
Mean	63.4	3.4	89.1	4.2	10195	3.5

Preop = preoperative, WNL = within normal limit.

## Discussion

Conservative therapy with sensitive antibiotics and bracing is adequate for most patients with infectious spondylitis. A delay in diagnosis and treatment is common in all forms of spinal infection because of their early indolent course [1-4]. Rezai et al. reported that 25% of patients who were initially treated nonsurgically had unsuccessful medial therapy [4]. Surgical intervention is typically reserved for patients with failed antibiotics therapy, intractable back pain, significant neurological deficit, large epidural abscesses, extensive vertebral body destruction, severe kyphotic deformity, or spinal instability [22-25]. However, major spinal surgery consisting of anterior debridement and bone grafting with or without supplemental instrumentation is often related to undesired postoperative complications.

Several minimally invasive methods have been used to treat infectious spondylitis. Computed tomography-guided percutaneous catheter drainage has been approved as an efficient and safe procedure in the management of early-stage spondylodiscitis [26]. Haaker et al. treated 16 patients with spondylodiscitis by using percutaneous lumbar discectomy. They concluded that it is a useful and minimally invasive technique for the conservative treatment of lumbar discitis, although the causative pathogens could be identified in only 45% of the cases [27]. Percutaneous suction, aspiration, drainage, and continuous irrigation with local administration of antibiotics have also been found to be effective in patients with early-stage pyogenic spondylitis and even spinal infection accompanied by iliopsoas abscesses [28-31]. However, the continuous irrigation method restrained the patients to their beds and limited their postoperative ambulation and activities.

The minimal invasiveness and simplicity of PED have led the authors to apply it as a modality for treating patients with infectious spondylitis. Direct endoscopic observation makes possible the direct collection of sufficient amounts of samples from the infected region for microbiological examination. Eradication and debridement of the infected and necrotic tissue from a disc and even an epidural space can be achieved under endoscopic monitoring. During the irrigation procedure, the disc debris and turbid abscess can be washed out by dilute betadine solution through the suction sheath. Moreover, postoperative negative-pressure Hemovac drainage in larger-diameter can continuously suck out the pathogens from the infected area. A combination of effective debridement with dilute betadine solution irrigation and full-course specific antimicrobial therapy resulted in favorable outcomes in current study.

Povidone-iodine is a widely used antiseptic and disinfectant agent. It can eradicate most pathogens, including oxacillin resistant staphylococcus aureus, and no bacterial resistance has been reported [14-18]. In an experimental research, Kaysinger et al. found that the inhibitory effect of betadine on embryo chick tibia and osteoblast cells is significant at concentrations of 5% betadine or higher [19]. In contrast, few cytotoxic effects were observed at a lower concentration (0.5% betadine). Goldenheim reported that 1%, 5%, or 10% povidone-iodine preparations do not have a deleterious effect on wound healing in animals and humans [20]. In a clinical study [32], spinal surgical wounds were soaked with dilute betadine solution before wound closure, and outcomes were compared with those of irrigation with normal saline. A 10% povidone-iodine solution was diluted to achieve a concentration of 3.5% betadine, possessing maximal bactericidal activity and minimal cytotoxicity. No wound infection occurred in patients who received betadine irrigation during the follow-up period.

Twenty-six patients with infectious spondylitis were successfully treated with PEDI in this series. This minimally invasive technique produces less morbidity than open surgery and provides effective relief to the patient’s back pain by reducing the intradiscal pressure and preserving adequate stability. These patients could ambulate with brace protection as early as possible after PEDI. Patients who sustained epidural or paraspinal abscesses could also be treated by this method and avoid having to undergo open anterior or posterior decompression surgery. A connection generally exists between these abscesses and the infected disc, which is the actual origin of spinal infection. After aggressive debridement of the infected disc and neighboring vertebral endplates, a cavity was created and even the sticky abscesses could be washed out by pressurized irrigation with betadine solution. With postoperative negative-pressure Hemovac suction, the pathogens in the infected tissue can be removed continually.

Six patients eventually underwent open surgery for poorly controlled infections and considerable mechanical back pain caused by progressive destruction. Three of 4 patients with multilevel infections who experience medical therapy failure received open anterior debridement and autograft interbody fusion with supplemental posterior fixation. Only 1 elderly patient with 2-level infections responded to PEDI. However, this patient still complained of mild back soreness with progressive kyphotic deformity. Therefore, the effectiveness of this procedure for extensive destruction of vertebral bodies and multilevel infections may be limited, from the viewpoint of either surgical technique or clinical prognosis.

This study has several limitations. First, we examined only 32 cases. Second, the retrospective nature of this study does not allow including patients undergoing different treatment methods for comparison, as well as lacks randomization because of ethical and legal considerations. The feasibility and benefits of PEDI for infectious spondylitis need to be rigorously evaluated in a large patient population with prospectively controlled comparison groups. Third, the enrolled patients had different kinds of spinal infections, such as early-stage infection or advanced infection, single-level infectious spondylodiscitis or multilevel infections, and spinal infection with or without epidural or paraspinal abscess. Thus, careful classification of patients through meticulous clinical examination and comprehensive image studies can further evaluate the efficacy and indications of this procedure in different stages and severities of spinal infection.

## Conclusions

On the basis of the findings obtained from this study, we propose that PEDI is an effective alternative to extensive open surgery for the treatment of single-level infectious spondylodiscitis or even complicated infection with epidural or paraspinal abscesses. It is successful in obtaining a bacteriologic diagnosis, relieving the patient’s symptoms, and assisting in the eradication of lumbar infectious spondylitis. Extensive anterior or posterior surgery is not always necessary in these cases. Additionally, the clinical course can be repeatedly evaluated and the treatment strategy can be modified accordingly after the PEDI procedure.

## Abbreviations

CRP: C-reactive protein; ESR: Elevated erythrocyte sedimentation rate; MRI: Magnetic resonance imaging; PED: Percutaneous endoscopic discectomy; PEDI: Percutaneous endoscopic debridement with dilute betadine solution irrigation.

## Competing interests

The authors declare that they have no competing interests.

## Authors’ contributions

SCY wrote the original manuscript, helped in the data analysis, and contributed to the interpretation and presentation of the data. TSF was responsible for the analysis and the interpretation of the data and contributed to all parts of the work of this study. HSC co-coordinated the study design, carried out the literature search, data analysis and drafting of the manuscript. YHK and SWY were responsible for the data acquisition, helped in the analysis and the interpretation of the data. YKT provided expert clinic advice in the field, design and structure and critically revised the manuscript. All authors read and approved the final manuscript.

## Pre-publication history

The pre-publication history for this paper can be accessed here:

http://www.biomedcentral.com/1471-2474/15/105/prepub
